# High output heart failure as a result of an ilio-caval fistula with a negative shunt series

**DOI:** 10.21542/gcsp.2021.16

**Published:** 2021-06-30

**Authors:** Travis R. Huffman, Nadine Sbaih, Mohammad Mathbout, Shahab Ghafghazi

**Affiliations:** 1 University of Kentucky College of Medicine, Bowling Green- Department of Cardiovascular Medicine, USA; 2 University of Louisville School of Medicine- Department of Internal Medicine, USA; 3 University of Louisville School of Medicine- Department of Cardiovascular Medicine, USA

## Abstract

Congestive heart failure (CHF) with high cardiac output is an uncommon, yet attributable result of non-hemodialysis arteriovenous malformations. While the prevalence of high output heart failure has yet to be determined, it is observably low - specifically when looking at cases of high output heart failure as a result of ruptured abdominal aortic aneurysms (AAA) with fistula formation, an entity that carries a reported incidence of <1% of all complications of AAA. In this report, we present a 64-year-old male with high output heart failure secondary to a ruptured right common iliac aneurysm causing right ilio-iliac and ilio-caval fistulas.

## Background

The purpose of this case report is to assist intensivists and cardiologists in recognizing high output heart failure as an entity, in which prompt diagnosis is crucial in order to provide the patient with the greatest chance at survival. It also emphasizes careful monitoring and thorough understanding of hemodynamics in patients with shunt-related high output heart failure, shunt repair, and subsequent right ventricle (RV) remodeling, as key elements in providing critical care for these patients.

## Case presentation

Our patient was a 64-year-old male with a past medical history of hypertension, infrarenal abdominal aortic aneurysm, and chronic obstructive pulmonary disease (COPD). He was on home oxygen and presented with abdominal pain, dyspnea at rest, and intermittent small volume hemoptysis. On admission, the patient was in acute renal failure, acute hepatic failure, and suspected acute decompensated heart failure, complicated by sepsis secondary to right lower lobe pneumonia.

He was treated empirically with broad spectrum antibiotics and underwent multiple imaging modalities, including CT pulmonary angiography, which showed no of evidence for pulmonary emboli. His abdominal pain was investigated with abdominal ultrasound, which identified a stable AAA without adequate visualization of the aortoiliac bifurcation. The patient failed to improve on broad spectrum antibiotics, and had negative blood, sputum and urine cultures. He deteriorated to hemodynamic collapse, requiring the addition of inotropes and vasopressors, with transfer to the intensive care unit (ICU) for management of suspected cardiogenic shock.

## Investigations

2D-echocardiogram showed an LVEF of 59%, remarkable only for moderate RV enlargement without notable anatomical abnormalities. A saline contrast study was negative for intra-cardiac and intra-pulmonary shunting. The patient underwent right heart catheterization (RHC), which identified a supra-physiologic mixed venous oxygen saturation of 86%, an elevated cardiac output of 14.88 L/min (calculated via Fick equation) and 10.89 L/min via thermodilution, with a calculated systemic vascular resistance (SVR) of 500 dyn/sec/cm^5^.

These findings were consistent with high output heart failure and decreased vascular resistance. A shunt series was performed, and was unremarkable for intra-cardiac/ pulmonary shunting, with a lack of step-up in hemoglobin oxygen saturation.

**Figure 1.**
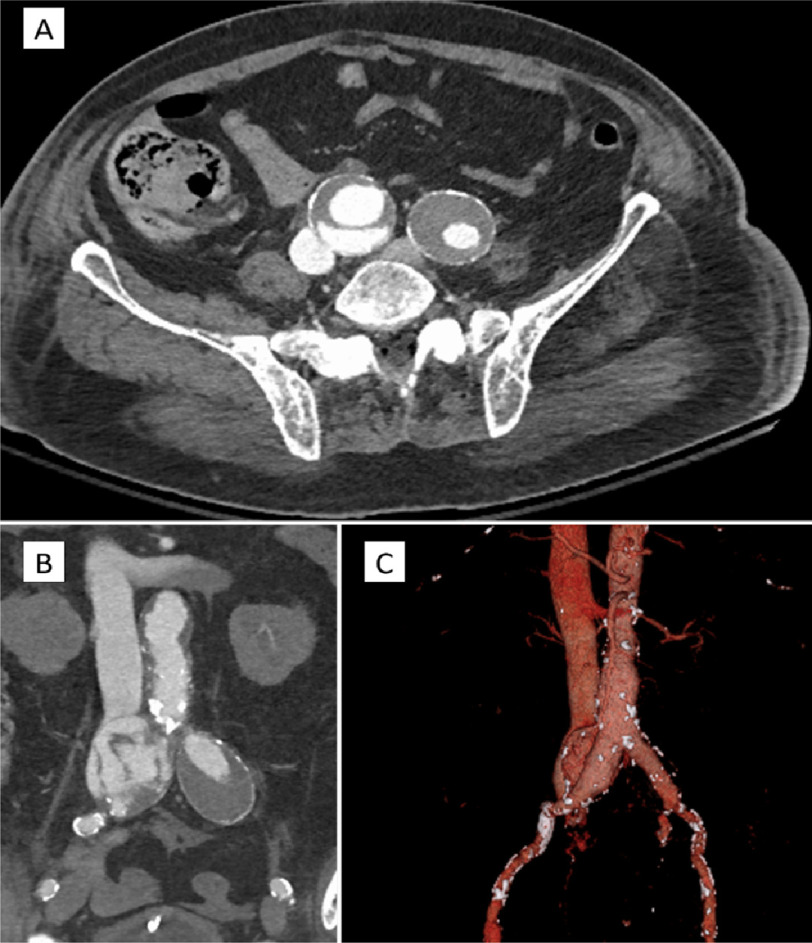
CT-Angiogram images demonstrating the fistulous communication between the right common iliac artery and the inferior vena cava. The axial (A), coronal (B), and 3D reconstructed (C), display the fistulous communication with radiographic dye traversing the defect. Images courtesy of the University of Louisville, Department of Radiology (Dr. Mohammad Ismail).

On subsequent physical examination, an abdominal bruit was appreciated on auscultation, prompting a CT angiogram of the abdomen and pelvis. CT identified a right common iliac artery aneurysm measuring 4.7 cm × 5 cm × 6.5 cm with evidence of recent rupture and fistulous communication with the adjacent right common iliac vein/caval confluence (Figure 1).

## Treatment

**Figure 2.**
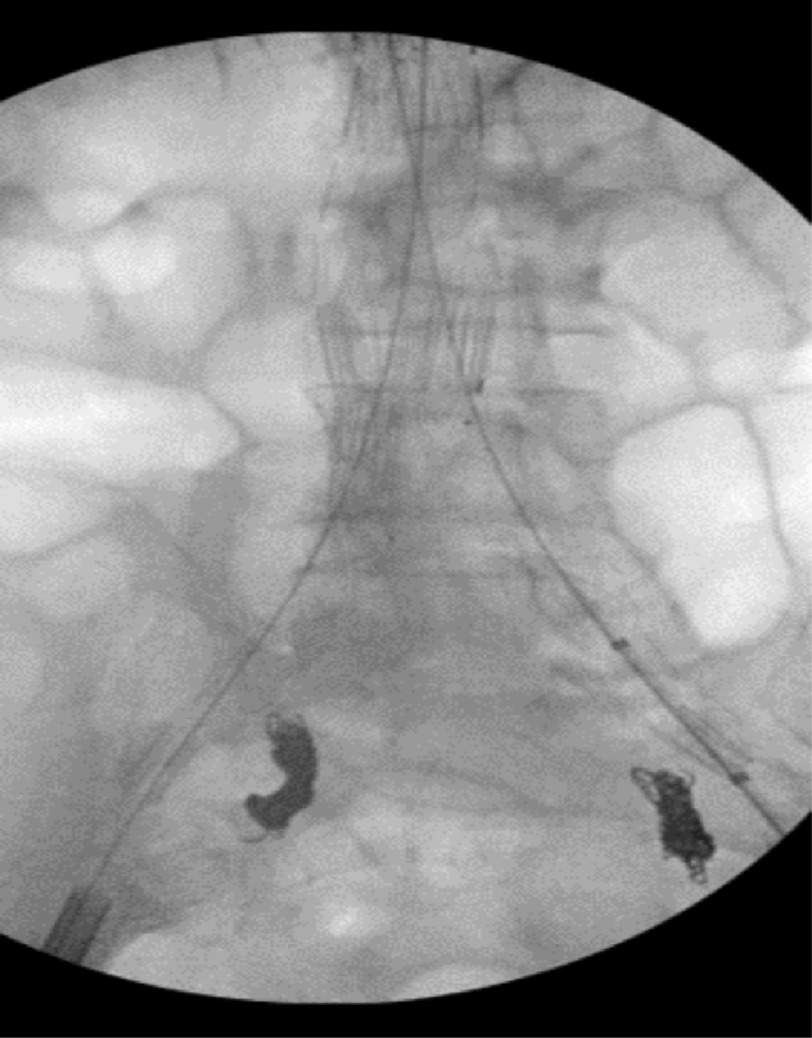
Intra-operative angiogram films obtained post bilateral coil embolization of the right and left common iliac artery, along with EVAR graft placement .

The defect was repaired by coil embolization of the right and left common iliac arteries, as well as endovascular (EVAR) graft to the main body of the infrarenal abdominal aortic aneurysm with extension into the right and left common iliac arteries. Intraoperative angiography showed marked reduction in trans-fistular blood flow. Post-operatively, anticoagulation was held with an expectation of false lumen thrombosis resulting in complete closure of the fistula (Figure 2).

## Outcome and follow-up

The patient was managed post-operatively with vasopressors and IV fluids to maintain a high central venous pressure (CVP)/RV pre-load. Over the next 3 days, his mixed venous oxygen saturation decreased from 86% to 69% and his cardiac output dropped from 14.88 L/min to 8.7 L/min. He was gradually weaned off vasopressors. He was also able to tolerate lower CVP and maintain his blood pressure.

## Discussion

High output heart failure as a result of a ruptured abdominal aortic/iliac aneurysm with resultant arteriovenous fistula is an uncommon entity, and one that carries a mortality rate approaching 90%^6,7^. In one system-wide review of high output heart failure, less than 1% (0.7%) (*n* = 120) of 16,462 consecutive patients who underwent RHC at the Mayo Clinic were diagnosed with definite high output heart failure based on criteria of elevated cardiac index in the setting of clinical heart failure as diagnosed based on Framingham criteria^1^.

Furthermore, of those identified with high output heart failure, only 22% (*n* = 27) had an arteriovenous (AV) fistula as the etiology of high output heart failure; 63% (*n* = 17) of these patients had an acquired fistula for the purposes of hemodialysis, while 30% (*n* = 8) were attributed to hereditary hemorrhagic telangiectasia (HHT). This leaves only 2 cases of non-acquired/non-congenital systemic arteriovenous shunt resulting in high output heart failure^1^.

Looking beyond the rarity of cases such as this, it is important to determine the etiology of high output heart failure, as treatment is, more often than not, only successful if the underlying etiology is identified and corrected. In this case, the differential diagnosis for the patient’s deranged hemodynamics included sepsis, liver disease, beriberi, and AV shunt/malformation. All etiologies were exhaustively investigated, and empirically treated, however hemodynamic derangements only improved after addressing the AV shunt.

We hypothesize that the shunt run performed during RHC was unremarkable due to the high-volume of blood traversing through the ilio-caval fistula. In other words, given cardiac output of 15 L/min, blood mixed very rapidly in the right atrium, hence, no identifiable step-up in oxygen saturations during.

This brings up the important clinical questions of the utility of RHC shunt series in the setting of large volume arteriovenous malformations, as well as the sensitivity of shunt series for extra-cardiac shunting in cases of high output heart failure without an identifiable etiology. The utility of shunt series to identify step-up/step-down in oxygen saturations or oxygen content are well documented in intra-cardiac shunts. However, with regards to extra-cardiac shunts, we were unable to identify any study that examined the sensitivity/utility of RHC with oxygen saturation sampling to identify systemic shunts likely due to rarity of this entity, with the exception of known acquired shunts, such as hemodialysis shunts and trans-jugular intrahepatic portosystemic shunt (TIPS) procedures^1–5^.

Another interesting aspect of this case was the post-operative management of this patient’s hemodynamics. Prior to correction of his AV fistula, it was unclear whether corrective surgery would result in RV failure as a result of the sudden drop in venous return/right ventricle preload. It appeared, in the immediate post-operative period, that this was the case, as the patient required increasing amounts of vasopressor support following surgery, that resolved with volume resuscitation and maintaining a high CVP. However, his blood pressure progressively improved over the course of several days, and we were able to wean off vasopressors and maintain an adequate blood pressure with lower CVP. It appears, in this case, that his RV remodeling was subacute, and that his RV was able to sustain the drastic change in end diastolic volume that occurred following corrective surgery.

Also playing a role in the management of this patients’ hemodynamics was the large change in vascular resistance that occurred after the AV fistula closed completely. Prior to correction of his vascular malformation, the patient had abnormally low systemic vascular resistance as a consequence of his large volume shunt. Surgical closure of the shunt then resulted in a sudden increase in systemic vascular resistance. However, in this case, although the shunt appeared to be reduced at the time of surgery, it was clear on the angiogram that closure was not complete. We utilized mixed venous oxygen saturations to evaluate the status and eventual complete resolution of his AV shunt. Interestingly, we noticed that as his mixed venous saturation declined (indicating resolution of his shunt), so did his RV pre-load and vasopressor requirements, in order to maintain mean arterial pressure (MAP) >65 mmHg. We did not observe any evidence of left ventricular failure or arterial hypertension.

### What have we learned?

• High output heart failure is an uncommon entity, and one in which prompt diagnosis is crucial in order to provide the patient with appropriate treatment and will directly affect survival.
• This case illustrates how the use of several modalities may be necessary to identify an AV shunt.
• Additionally, this case illustrates how careful monitoring and thorough understanding of hemodynamics in shunt-related high output heart failure, shunt repair, and subsequent RV remodeling, can aid the management of these patients.
